# The collective lie in ketamine therapy: a call to realign clinical practice with neurobiology

**DOI:** 10.3389/fpsyt.2025.1610335

**Published:** 2025-09-22

**Authors:** Charles Miller, Bryan Lopes, Anna McCurdy

**Affiliations:** ^1^ Scenic City Neurotherapy and Ketamine Center LLC, Chattanooga, TN, United States; ^2^ RailRoad Valley Therapeutics LLC, Chattanooga, TN, United States

**Keywords:** ketamine, neuroplasticity, psychoplastogen, treatment-resistant depression (TRD), ketamine-assisted psychotherapy (KAP), NMDA receptor antagonist, evidence-based psychiatry, psychedelic therapy

## Abstract

In recent years, ketamine therapy has become increasingly entangled with psychedelic culture, leading to widespread misinterpretation of its therapeutic mechanism. This manuscript challenges the prevailing narrative that positions ketamine as a consciousness-expanding agent or psychotherapy enhancer, highlighting the discord between this view and ketamine’s well-established neurobiological function as an N-methyl-D-aspartate (NMDA) receptor antagonist that promotes neuroplasticity. Drawing on recent research and clinical data, the article argues that the acute dissociative experience often emphasized in ketamine-assisted psychotherapy (KAP) is neither necessary nor sufficient for therapeutic success. Instead, it describes how meaningful, lasting improvement in mental health outcomes requires plasticity-driven reorganization in the days following ketamine administration, not from insights gleaned during dissociation. By prioritizing subjective experience over biological timing, current KAP practitioners risk distorting memory, reinforcing maladaptive narratives, and undermining the potential of psychoplastogenic treatments. This article calls for a shift toward evidence-based protocols that align clinical practice with neurophysiology, advocating for greater education, standardization, and scientific rigor in ketamine therapy.

## 1 Introduction

Upon entering this field in 2019, the expectation was to find a community grounded in neuroscience and psychiatry—one driven by solid data, peer-reviewed research, and evidence-based protocols. Instead, much of the ketamine conversation and emerging landscape was shaped by personal interpretation and experiential enthusiasm rather than clinical evidence. It was surprising to find non-medical providers speculating about ketamine’s potential as a psychedelic, often without reference to its well-documented mechanism as an N-methyl D-aspartate (NMDA) receptor antagonist that promotes neuroplasticity through specific physiological processes established by preclinical and human studies (1–5). An unyielding belief in the transformative power of the ketamine experience has allowed for a divergence of optimal administration methods. The desire to democratize ketamine therapy has led to compromises in administration, doses, and durations of treatment. This modification has diminished the therapeutic potential of ketamine in the treatment of depressive disorders (17).

## 2 History of the lie

Despite an ever-growing knowledge base on the mechanisms of ketamine and neuroplasticity, which began with the foundational ketamine research from Yale in the early 2000s (6, 7). Many of our current programs that offer continuing education on the therapeutic use of ketamine in the treatment of depressive disorders will downplay the role of neurobiology. Most of these programs will focus instead on the subjective experiences reported by a patient under ketamine’s influence. Practitioners who subscribe to the experientially driven transformative power of the ketamine belief system have begun using terminology far removed from the clinical lexicon. In some cases, medical terms have been reinterpreted or reinvented. An example of terminology reinvention is the term “empathomimetic,” which serves as a mechanical classification for ketamine’s ability to stimulate empathy and self-reflection. This pseudoscientific descriptor is derived from an actual pharmacologic drug classification, “sympathomimetic.” This influx and adoption of pseudoscientific language is contrary to existing medical terminology. This abandonment of objectivity is reflected in the subjective inferences surrounding the use of therapeutic ketamine’s shift toward symbolic language over scientific clarity (8, 9). This alternative dialogue legitimizes the practitioners’ belief system, despite the lack of empirical evidence, as the foundation of their claims relies on unquantifiable variables, speculation, and anecdotes.

In the 2010s, dozens of ketamine infusion centers opened across the United States and many countries worldwide. Medical and non-medical providers were excited about the potential of ketamine and desired to participate in the care and administration of this novel therapy (10). Podcasts and popular culture reignited the conversation of psychedelic therapies. Through continued dialogue, social media posts, and online advertising, pop culture’s ignorance of ketamine as a psychoplastogen led to rampant speculation and psychedelic enthusiasm. Fueled by misinformation from seemingly reputable scientific outlets, the greater medical community interested in the use of therapeutic ketamine slowly began to adopt the belief that the dissociative state was, in fact, the treatment goal. This alteration in therapeutic goal from the stimulation of neuroplasticity to facilitating a transformative experience allowed for compromises in the route, dose, and duration that would demonstrably diminish Brain-Derived Neurotrophic Factor (BDNF) release and subsequent neuroplasticity response (11).

## 3 Perpetuating the lie

Everyone has had moments when they experience the feeling of clarity or deep understanding. Sometimes that feeling is well-founded due to knowledge gained. Other times, “the lack of expertise and knowledge often hides in the realm of the ‘unknown unknowns’ or is disguised by erroneous beliefs and background knowledge that only appear to be sufficient to conclude a right answer” (12). This phenomenon is called the Dunning-Kruger Effect. Psychedelic or dissociative experiences can mimic this phenomenon, but they do so under very different neurobiological conditions than we experience in our sober state. Under the influence of ketamine, the brain enters a state of reduced inhibition and heightened suggestibility (13). This combination, not unlike what we observe with substances like alcohol or serotonergic psychedelics, can produce a subjective sense of emotional openness or epiphany (14). However, these effects are not indicative of improved cognitive processing or heightened understanding but are instead the result of ketamine’s mechanical disruption of neural connectivity.

Confusion between therapeutic ketamine and psychedelic pseudoscience poses a significant risk to the legitimate use of psychoplastogens in treating mental health, chronic pain, and neurocognitive disorders. The growing influence of psychedelic advocacy groups, “thought leaders”, and integrative practitioners has created an environment where narratives and beliefs often eclipse mechanisms. Organizations such as the Multidisciplinary Association of Psychedelic Studies (MAPS) and the American Society of Ketamine Physicians, Practitioners, and Psychotherapists (ASKP3), while advancing awareness, have also contributed to the self-reinforcing misinformation systems that prioritize the subjective experience. This shift has begun to obscure the measurable and mappable effects that psychoplastogens can have on the brain, reducing their legitimacy and potential in the eyes of skeptical clinicians and researchers.

Today, patients are often introduced to ketamine therapy by physicians or advanced practitioners who are unaware of ketamine’s stimulation of neuroplasticity or how to provide patient education so they can leverage the post-treatment period for cognitive and emotional development. Instead, ketamine, like serotonergic psychedelics, is frequently framed by the provider as a tool for enhancing the psychotherapy itself (15). The belief that ketamine can catalyze insight or expand consciousness has become central in many treatment narratives, such as in ketamine-assisted psychotherapy (KAP), despite the lack of evidence supporting an experiential mechanism for lasting change. The ability of ketamine to stimulate neuritogenesis, synaptogenesis, and plasticity in maladaptive synaptic processes, as well as observable improvement in global connectivity, makes ketamine unique among other psychoplastogens (16). Literature comparing administration modalities such as intravenous, intramuscular, sublingual, and intranasal reinforces the existing knowledge base regarding bioavailability and therapeutic efficacy (11, 17). Intravenous administration of ketamine promotes optimal symptom reduction for greater periods when compared to other routes of administration. This much is well established despite the ever-expanding knowledge base regarding the mechanisms of psychoplastogens.

## 4 Fact vs. feelings

People will struggle with objective self-reflection due to inherent cognitive biases even in baseline consciousness. Neural lesions and alterations can lead to diminished cognitive processing and functional neural connectivity (18). Psychoplastogens stimulate the formation of new neural pathways and the reorganization of synaptic pathways (19). In the case of ketamine, this process does not occur during the acute dissociative experience, as is observed with serotonergic psychedelics. Instead, it unfolds in the hours and days following treatment, primarily during rest and sleep (2, 20, 21). Ketamine blocks NMDA receptors, which triggers a glutamate surge, activating α-Amino-3-hydroxy-5-methyl-4-isoxazolepropionic acid (AMPA) receptors. This inhibition of NMDA and stimulation of AMPA leads to the release of BDNF and downstream mammalian target of rapamycin (mTOR) signaling. The preclinical research suggested that the optimal neuroplastic window starts to open once the drug has cleared the system, which can be hours later (11, 22, 23). In short, the vivid thoughts and emotions experienced during a ketamine session are a transient, pharmacologically driven phenomenon, much like a dream. When dreaming, theta brain wave states are remarkably similar to the observed theta brain wave states produced by ketamine (24).

Framing ketamine’s dissociative state as a psychotherapy enhancer misrepresents its action and can limit its potential benefit. At best, therapy conducted during the altered state may function as a form of chemically induced hypnosis, temporarily shifting patterns of thought. At worst, exploring trauma during these states of high suggestibility can lead to the reinforcement of maladaptive narratives or even the introduction of new distortions. While the intentions behind this approach are often compassionate, we must be clear-eyed about the risks: re-traumatization, memory distortion, and disruption of neurobiological processes essential for long-term healing.

Patient education needs to reflect how positive mindset shifts can emerge from the acute treatment experience, but they should not be mistaken for a truth that promotes durable change. Plasticity begins when the inhibition of neuronal communication subsides, and the brain is at rest (21). Functional plasticity is the first step toward improved cognition, functionality, and emotional regulation. The allocation of dendritic pathways in the synaptic network occurs in the days, weeks, and even months following ketamine’s initial stimulation of plasticity by optimizing the patient’s daily life practices and work, supported by a licensed therapist. The meaningful and lasting benefit comes from reflection and behavioral reinforcement that occurs in the period following neurophysiological optimization. It does not result from a transient dissociative experience (21). The plasticity window begins after ketamine’s effects have fully subsided and lasts much longer than the window observed with serotonergic psychedelics (18, 23, 25, 26). Informed consent demands that patient education include an explanation of the role of plasticity as well as the value of the thoughts and feelings experienced while under the influence of ketamine. Helping the patient understand that profound feelings or transformative experiences during acute administration are not a requirement for long-term benefit. Instead, the value of the perceived experience is like that of a dream: symbolic, emotionally charged, but not inherently instructive, or even truthful. Providers should not overemphasize the meaning of the acute experience or create an expectation that lasting transformation occurs within the session itself.

## 5 Conclusion

Neuroplasticity is not just some vague state of openness—it relies on a biologically timed window and allows the individual to create and allocate new behavioral and reactivity pathways. Approaching ketamine administration and management as if it were a psychedelic ignores the well-understood mechanisms of the drug. The window for serotonergic psychedelics like psilocybin is the result of a strong activation of 5-hydroxytryptamine 2A (5-HT2A) receptors, which directly modulate cortical pyramidal neurons and stimulate immediate synaptic reorganization (19, 27). Therefore, in those sessions, a rationale for therapeutic framing or direction during the acute experience may exist. Even still, the therapist must navigate the potential risks of reinforcement of trauma or maladaptive narratives that are high in altered states (9).

But ketamine? It works differently. The primary driver of plasticity is not immediate dissociation—the neurobiological cascade occurs once ketamine’s effects have subsided. Even then, plasticity is not the corrective mechanism but rather the first step in rebuilding adaptability, improving function, and adjusting emotional responsiveness to stimuli. Applying deep psychotherapy during peak dissociation is like planting seeds in frozen ground. The conditions are not quite right.

Therapeutic efforts should focus on elevated plasticity during the days following treatment, when intentional practices can reinforce new connections. The goal is not to recreate the altered state, but to help patients build sustainable patterns of thought and behavior. Framing success in terms of dissociative depth or peak emotional experience risks encouraging dependency on the compound or experience, rather than promoting agency and resilience.

Ultimately, successful treatment requires clarity; clarity about what ketamine can and cannot do, and about when and how change truly happens. When we align our protocols and the essential psychotherapeutic interventions with the brain’s actual timeline, rather than the traditions of psychedelic culture or the expectations of immediate insight, we offer our patients something far more potent than a momentary shift. We offer them the groundwork for enduring transformation.

## Data Availability

The original contributions presented in the study are included in the article/supplementary material. Further inquiries can be directed to the corresponding author.
